# Characteristics of Early Phase Clinical Trials for Rare Cancers: Insights From Interviews With Stakeholders

**DOI:** 10.3389/fphar.2022.775217

**Published:** 2022-05-02

**Authors:** M Dooms, R Saesen, I Steemans, J Lansens, I Huys

**Affiliations:** Department of Pharmaceutical and Pharmacological Sciences, Faculty of Pharmaceutical Sciences, Clinical Pharmacology and Pharmacotherapy Research Group, KU Leuven, Leuven, Belgium

**Keywords:** rare cancer, phase I clinical trials, medical oncology, first in human, orphan drug, basket trial

## Abstract

**Background:** Rare cancers occur with an incidence of no more than six cases per 100,000 people according to the definition used by the Surveillance of Rare Cancers in Europe project. For a variety of reasons (low prevalence, cytotoxicity), it is challenging to perform the necessary clinical studies to investigate the safety and efficacy of investigational medicines against such rare malignancies, reformulating even at the earliest stages of the drug development process. This article investigates the differences between phase I rare cancer trials performed in commercial (companies) and non-commercial settings (academic hospitals).

**Materials and Methods:** The differences were explored through the conduct of semi-structured interviews with three different stakeholder groups: representatives from academia (*n* = 7), representatives from companies (*n* = 4) and representatives from patient organizations (*n* = 4). All the interviews were transcribed verbatim and analyzed in NVivo using the framework method.

**Results:** According to the interviewees, the academic and commercial stakeholders collaborate in the majority of phase I rare cancer trials. In general, the commercial partner finances the trial, whereas academia is responsible for the execution of the study procedures. The average cost of undertaking these trials is difficult to estimate because it depends on what is specifically requested during the trial. The 3 + 3 study design remains the most widely used design and the use of expansion cohorts is controversial. With regard to the regulatory aspects of phase I rare cancer trials, it was expressed that a good regulatory framework facilitates the conduct of these studies, but that increased regulation and oversight also has drawbacks, e.g., differences in standards between different ethics committees, over interpretation of the rules, insufficient availability of qualified personnel and higher workloads. The patient organization representatives claimed that patients experience no differences in terms of accommodation, compensation and paperwork between the academic and commercial settings or the degree of follow-up. They also believed that the direct input of patients can bring added value to such studies not only with regard to the recruitment process and the feasibility of the study but also the legibility of the informed consent forms.

**Conclusion:** The growing need for first-in-man trials in rare malignancies needs to be highlighted, as difficult as they are to undertake and to co-develop, not only because rare cancer patients deserve an appropriate treatment, but also because these medicines represent the future of cancer therapy in the precision medicine era. Cooperation of commercial and academic sites are needed. Patient organizations need to be educated to take part in this process.

## Introduction

### Rare Cancers

#### Definition of Rare Cancers

The definition (Gatta et al., 2011) of rare cancers is not internationally standardized. According to the Surveillance of Rare Cancers in Europe (RARECARE: http://www.rarecare.eu/) project, the incidence of a malignancy should be no higher than 6 cases per 100,000 people per year to be considered rare. This implies that fewer than 30,000 new rare cancer cases are diagnosed in Europe every year.

Nevertheless, since there are 186 different types of rare cancers known (Gatta et al., 2011), their disease burden is high, amounting to 22% of all cancers in the European Union, affecting more than 4.3 million citizens. Moreover, this is likely still an underestimation (Komatsubara and Carvajal, 2016) of the incidence of these diseases because of the change in classification of cancers over time from a histological to a molecular-based taxonomy. In the histological classification (Boyd et al., 2016), tumors were classified as rare on the basis of satisfying one of two definitions. The first definition considers a tumor rare if it originates from cell types that do not often cause cancer. The second definition states that rare tumors are histologically defined subgroups of more common cancers. In the molecular-based classification (Boyd et al., 2016), which is used most often nowadays, tumors are rare if they have a distinctive histology and underwent a molecular alteration, such as mutations or other genomic aberrations. Tumors are also described as rare if they only have molecular alterations. Because of this shift in the definition of rare cancers, they are diagnosed more often today and the number of cases will therefore in all likelihood continue to rise over time.

More advanced research efforts also contribute to the rising number of rare cancers, as cancers that used to be classified as common are now becoming rare due to the development and use of diagnostic tools based on the detection of genetic mutations (Billingham et al., 2016). There is a clear and rising need for accurate methods to assess the safety and efficacy of novel clinical interventions against these malignancies due to the emergence of more personalized treatments and the increasingly detailed molecular characterization of cancers.

#### Treatment of Rare Cancers

Although there is often a lack of therapeutic options for rare cancers, the available treatments do show very high response rates (Olver, 2016). The reason for this is that rare cancers typically display less patient variability in genetic mutations compared to common cancers. As a result, treatments for rare cancers, if they are available, are more precise and targeted to the mutation. At present, the European Medicines Agency (EMA) has granted marketing authorizations to 205 orphan drugs, of which 61 were indicated for rare cancers (Wirth and Ylä-Herttuala, 2014; Kato et al., 2015; EMA, 2018; Ginn et al., 2018). Only 36 of these went through a first-in-man clinical trial before authorization as the other 25 were repurposed.

#### Challenges of Rare Cancers

The diagnosis and treatment of rare cancers are often suboptimal due to limited knowledge and expertise on the part of treating physicians (Gatta et al., 2011). As a result, the survival rate (Olver, 2016) of rare cancers is lower than the survival rate for more common ones. It is therefore important that an improvement in diagnosis and treatment of rare cancer care (Gatta et al., 2011) is established. One of the suggested solutions is to centralize care at specialized centers When further examining the late diagnosis of most rare cancers (Blay et al., 2016), this can be explained by low diagnostic precision, which is determined by the awareness, experience and competence of the medical team. A lack of diagnostic precision can also result in the mismanagement of care. All these challenges increase the overall burden of rare cancers (Gatta et al., 2011). This pinpoints to an urgent need for new and effective treatments for rare cancer patients. To address these challenges, a European partnership has been established under the name Rare Cancers Europe (RCE: https://www.rarecancerseurope.org/) (Casali et al., 2015).

### Phase I Clinical Trials

Clinical trials (Dooms, 2017) are conducted to evaluate the safety and/or the efficacy of (investigational) medicinal products developed for a specific disease. Phase I trials (“First-in-Human,” “Early phase”) are the first studies performed in humans and explore the optimal dose, the safety and the tolerability of the investigational drug. In general, phase I clinical trials are conducted in a small group of healthy volunteers. Exceptionally, these First-in-man studies can also be conducted in patients. An example of a field in which this occurs is oncology (Ursino et al., 2017), given the potential cytotoxicity of these drugs. For gene-editing products (Dooms, 2017), it is also difficult to conduct a phase I clinical trial in healthy volunteers since doing so would also be considered unethical.

#### Challenges of Phase I Clinical Trials for Rare Cancers

To conduct a robust clinical trial, a sufficient number of patients needs to be enrolled. This already creates one of the first major challenges for rare cancer clinical research, since the number of patients is very limited (Casali et al., 2015).

One of the potential ways to address this challenge is through increased international collaboration and making use of the European Reference Networks EURACAN (https://euracan.eu/) and PaedCan, (https://paedcan.ern-net.eu/), which will boost the number of patients eligible for recruitment. However, such collaborations also pose a few additional challenges (Komatsubara and Carvajal, 2016). Firstly, reaching a consensus about the design and management of the trial is often a problem in practice, as approaches thereto can differ from region to region. Additionally, there are differences in clinical research laws and regulations between countries, although these may be addressed by new Clinical Trial Regulation. A third problem is a logistical one and relates to the distribution of resources/facilities between study centers as we study rare conditions. Lastly, the difficulty with separating different cancer types into even smaller subgroups (Blay et al., 2016) is that it can lead to an increase in the number of clinical trials needed. Considering the high costs associated with conducting clinical trials today, there is often a lack of sufficient funding available for such trials, and multiple outside sources of funding therefore need to be combined. All of these challenges indicate that new methodologies are needed for which a smaller number of patients have to be recruited.

#### Methodology of Phase I Clinical Trials for Rare Cancers

##### Traditional Trial Designs

One of the most widely used study designs in phase I oncology trials is the 3 + 3 design (Le Tourneau et al., 2009). In this design, three patients are initially treated with a safe dose that is based on toxicological data derived from studies in animals. If none of these patients experience toxicity, three additional patients are given a slightly higher dose for a certain period of time. This cycle continues and the dose progressively increases until two of the three patients within a cohort experience dose-limiting toxicities. However, if only one of the three patients experiences toxicity, then three new patients are treated with the same dose. The dose that is considered the right dose is the dose just below the toxic threshold.

This study design is considered a safe method for finding the right dose for the subsequent phase II studies. Another advantage of this design is that it generates information about pharmacokinetic variability. A remark that has to be made here is that within this approach, many patients are treated with a low and perhaps even sub therapeutic dose.

However, at present, phase I clinical trials are usually not only looking to produce safety or pharmacokinetic data. By adding dose expansion cohorts (Iasonos and O'Quigley, 2015) (i.e., additional groups of patients) to early phase studies, efficacy can be determined at an early stage and the most promising drugs can be singled out. This can save sponsors time and money down the line and such an approach will be advantageous for patients as it speeds up time to orphan drug authorization.

Another traditional early phase trial setup is the rolling six design. This study design (Doussau et al., 2016) has a similar approach to a 3 + 3 design. Here, six patients are treated with the same dose. To find the next dose which the following patient cohort will be treated with, a number of different factors are considered, including the number of participants enrolled at that moment, the number of participants that experience toxicity, and the number of patients who are being screened for participation. A decrease in dose is applied when two or more patients experience toxicity at a certain dose level. Otherwise the dose will be increased.

##### New Trial Designs

To tackle the challenges (Renfro and Sargent, 2017; West, 2017; Woodcock and Lavange, 2017; Park et al., 2019) accompanying the conduct of clinical trials in rare cancers, new types of trials have been designed, namely the umbrella, basket and platform trials. The main advantage (Park et al., 2019) of these new trials designs is that they can be adapted depending on the research objectives and the indications of interest.

*The umbrella trial (West, 2017) divides patients into groups with the same basic cancer type. Afterwards, molecular marker tests for different potential targets are carried out. Based on the presence of a mutation matched to a potentially effective treatment for that marker, the patients are assigned to different arms of the study. In some cases, the presence of a specific marker does not have to be tested and patients are randomized to a “default arm” consisting of a treatment strategy with broad activity. During the study, arms can open or close when the trial is modified based on the emergence of new targets or treatments.
*Basket trials (West, 2017) include patients who have the same genetic driver mutations, but different tissues or organs of origin. These patients are given the same novel treatment with the specific marker that they all have in common as a target. The experimental treatment is therefore administered based on the mutations underlying the tumor instead of its tissue or organ of origin.
*Platform trials are also called the multi-arm, multi-stage design trial (Park et al., 2019). By using this trial design, a multitude of interventions can be tested and compared to a control group. Rules for adapting the trial protocol are formulated prior to the start of the study. These rules ensure that ineffective treatment options can be dropped and that new interventions can be added. This implies that the research question can change over time based on new data that becomes available. This trial design ensures research can be done more efficiently.

Besides these new study designs, Bayesian methods (Berry, 2006) are also used more and more in clinical trials because they allow adaptation of the study design based on information that becomes available during the trial.

*A Bayesian clinical study design (Pallmann et al., 2018) continually calculates the probability distribution for certain outcomes based on changes in the data. Because of this, it can combine and assess newly available data together with already existing data. This also means that the investigator can make clinical decisions (Casali et al., 2015) during the trial based on the probability distribution. This statistical method is becoming more popular in phase I trials and is also being implemented into umbrella and basket trials.

## Materials and Methods

The objective of this paper is to determine the important differences and possible cooperation between non-commercially funded phase I rare cancer trials executed in a non-commercial setting (“*academic*”) and commercially financed early phase rare cancer studies conducted in a commercial/non-commercial setting. The different aspects were explored through semi-structured interviews with relevant stakeholders. No quantitative data were collected. Instead, qualitative information such as opinions, remarks, concerns and thoughts of experts and patient organizations were collected. No existing contracts between the sponsor and the trial center were examined.

Purposive (dedicated institutions) and snowball sampling methods were used to select the study sample. Three different groups of participants were interviewed. The first two groups were clinicians (“academics”) involved in a non-commercially funded early phase clinical trial for rare cancers conducted in a non-commercial setting and clinicians involved in a commercially funded early phase clinical trial for rare cancers conducted in a non-commercial/commercial (“institunional”) setting. These groups were interviewed to gain insight into the organization of these clinical trials and to investigate the perceived differences between these two settings. The third group of interviewees was composed of representatives of organizations for patients with rare cancers. They were interviewed to further understand the patient’s perspectives on the differences between the two groups mentioned above, as well as to find out how patient organizations are involved in these trials. Inclusion criteria for clinicians were that they had to be involved as an active investigator in a phase I clinical trial for rare cancers in adults within Europe. Representatives of patient organizations needed to represent a European (rare) cancer patient organization and were contacted during meetings, calls, courses, consultations and the like.

This study ran from the first of September 2019 until the end of March 2020.

Participants fitting the inclusion criteria were selected based on their expertise as well as on suggestions made by the interviewees themselves. Next, the candidates were invited to participate by e-mail. If they showed interest to participate in this study, they were sent an informed consent form mentioning the practical details surrounding the study and explaining its objectives. Once they had returned the signed consent form, an interview was planned and conducted. As participants were working in different European countries, the interviews were conducted *via* Skype^®^. The interview session was recorded using Skype’s^®^ built-in recording function, which the participants were aware of and had agreed to by signing the informed consent form. On average, an interview took about 45 min. After the interviews had been conducted, they were transcribed verbatim. Once this was completed, the interview recordings were deleted and the transcripts were qualitatively analyzed in NVivo exclusively using the framework method (Gale et al., 2013). Reading and coding was done by two students and one experienced researcher. The themes emerged from an extensive literature search.

## Results

### Research Sample

In total, 87 possible participants were contacted, of whom 15 agreed to take part in the study and were subsequently interviewed.

Because this study focused on the European setting, participants from different European countries were included. The representatives from the commercial side included employees of the medical department of large pharmaceutical companies (i.e., international companies that have offices all over the world). For the academic side, clinical oncologists from five different EU countries were included.

### Setting

From the interviews, it became clear that phase I clinical trials for rare cancers are almost never executed in the clinical trial units of the pharmaceutical companies but generally in academic centers with experienced investigators and study nurses/pharmacists, responsible to perform the trial as well as patient care. Bearing in mind that rare cancer patients are often taking multiple concomitant medicines, the guidance of these patients by (clinical) hospital pharmacists is important. Another reason why these trials are generally performed in a hospital setting is that they often require extensive resources (e.g., MRI, CT scans, bone marrow punctures), which are readily available in an academic hospital setting.

An important remark made is that early phase clinical trials are often not specific to one cancer type. The study population often includes both common and rare cancer patients.

### Financing

#### Funding Mechanisms

Three different mechanisms of financing phase I clinical trials for rare cancers emerged from the interviews. The vast majority of studies are financed by pharmaceutical companies. In most cases, a pharmaceutical company has developed an investigational (orphan) medicinal product that they want to test in patients. To do so, they seek contact with academic centers and negotiate the needed budget with them. Representative 2 from the commercial side noted that they often cooperate with the same academic centers: “*We always try to start from previous experience in the hospital, because we try to have some fixed costs, like the MRI and CT.”* Secondly, representatives 4 and 6 of the academic side claimed that in rare cases the trial can also be co-financed by the academic center itself. However, support from the pharmaceutical industry is still needed in this case, mostly because of the limited budget of the academic centers. A third, but very rare, possibility is that an early phase trial for rare cancers can also be fully financed by independent organizations or with public means. This possibility was raised by representatives 2 and 5 from the academic side. For example, the KCE Trials Program in Belgium (https://kce.fgov.be/nl/kce-trials) can do that but has so far not yet funded any phase I clinical trials.

#### Costs of Execution

Most of the participants stated that the specific budget assigned for the execution of these clinical trials is difficult to estimate. For example, representative 2 of the commercial side said the following: *“The costs really depend on what is requested in that trial. For example, if you need a lot of MRIs and a lot of bone marrow biopsies, then the price will immediately increase.”* Based on the pooled statements of all the interviewees, it can be estimated that the amount ranges between 10.000 and 50.000 euros per patient, but this is certainly not always the case.

#### Participant Compensations

All interviewees asserted that the patients who enter a First-in-Man clinical trial for rare cancers are not compensated for their participation, unlike the healthy volunteers taking part in phase I studies for other indications. Representative 4 of the academic side stated: “*That is because we really want to avoid the situation that patients are participating in trials because of the income that it produces. So, it has to be a free choice*.” Paying the patient for participating is not seen as ethical. However, participants do get compensated for the costs associated with their participation in the trial (e.g., overnight stays in a hotel, transportation to the hospital, parking costs) by the party funding the trial.

### Regulation and Oversight

#### Inspections and Audits

During the interviews it became clear that the way in which internal and external audits and inspections are conducted in the course of a phase I clinical trial for rare cancers is generally the same all over Europe. Nevertheless, their frequency varies greatly.

While the frequency of inspections varies, in general it can be stated that they occur between once every year and every 3 years. For example, representative 2 from the commercial side said that they occur once a year, while representatives 4 and 7 from the academic side mentioned that they take place every 2–3 years.

The audits can be divided into two groups: the internal audits and the external audits.

The internal audits are organized by the company/hospital itself. Both the representatives of the academic and commercial sides mentioned that they happen very often. Representative 1 of the commercial side made the following statement in this regard: *“So the internal audits, we do them very frequently. At least 2 times a year, so that we have this constant monitoring of quality. Not only through inspections, but also through regular trainings, learnings, compliance sessions et cetera.”*

External audits are organized by the sponsor, so most of the time by pharmaceutical companies. Their intensity varies, but a general trend was observed. *“It all depends on your recruitment. So, the higher the recruitment, the higher the chance you get an audit,”* representative 2 of the academic side claimed.

#### Positive Regulatory Aspects

First, four interviewees claimed that some European countries have a more favorable regulatory environment for conducting early phase clinical trials for rare cancers, for example because of faster approval times of study protocols by local ethics committees. Representative 1 of the commercial side stated for Belgium in particular: “*We have an authority that secures a very quick turn-around. This turn-around between submission and approval by regulatory authorities is 2 weeks, which is extremely competitive when we compare it to other countries.*” This advantage sets Belgium apart from other European countries, and is therefore seen as an attractive country to execute clinical trials.

A second positive regulatory aspect that was mentioned is the Clinical Trial Regulation (Regulation (EU) No. 536/2014, expected to come into application soon), which was composed with the aim of creating a favorable environment for the execution of clinical trials in the European Union and ensuring a more uniform interpretation and application of laws pertaining to clinical research across Europe. Thirdly, it became clear from the interviews that the possibility to request early scientific advice by regulators was considered very helpful. Representative 1 of the commercial side stated the following: “*Whenever we do a phase I trial, we always seek the opportunity to engage with regulatory authorities to address their questions and to address the concerns that we have before we set the trials up.”* This gives the trial sponsors the opportunity to improve the quality of the study.

In general, the interviewees highlighted that First-in-Man clinical trials for rare cancers are heavily regulated. They highlighted the necessity of regulatory measures to ensure the protection of the participants. For example, representative 7 of the academic side claimed that “*the rules are necessary because you have a group of patients who does not have standard options anymore or does not have standard options at all. So you have to protect patients from mistreatment by sticking to the rules.*”

#### Challenges at the Regulatory Level

Representative 7 of the academic side claimed that every hospital has its own ethics committee and that this is accompanied by a number of challenges. The first one is that the decision-making process can take a very long time, as the members of the committees are often clinicians who have to combine their assessment of trial protocols with their usual day-to-day clinical work.

The second challenge relating to the ethics committees of different hospitals is that there is little harmonization between them. This makes it difficult to know which data are needed for each committee. If there could be more standardization of the data that have to be submitted, it would simplify the process of submitting a trial protocol to the ethics committee. This last challenge was also cited by representative 4 of the commercial side.

As mentioned above, the laws and regulations pertaining to the conduct of clinical trials need to be respected. However, there were some comments from interviewees about the interpretation of the rules. To illustrate this, the example of informed consent forms was mentioned. These documents are needed to inform the patient, but the interviewees lamented the fact that newer versions are often released and that these need to be signed again by the participants. Representative 5 of the academic side mentioned the following in this regard: “*The interpretation of the rules makes it very difficult to perform these trials.”*

To set up a phase I clinical trial, you not only need experienced study staff, but also a sufficient number of people coordinating the trial and communicating with the regulators. If you have a lack of personnel, the organization of a clinical trial can be very challenging, but if you have these human resources, it becomes more feasible. Representative 3 from the commercial side stated the following: *“The burden of the regulations depends on the quality of the regulatory affairs people; how much experience they have and how good they are in the communication with the agency.”* The problem is often that qualified study personnel are insufficiently available, which results in clinicians also having to do the regulatory work, while they ideally want to spend as much time as possible on providing care to the patients.

The trial execution itself also demands great efforts from health care providers. To illustrate this, representative 3 of the commercial side made the following remark: “*You are responsible, so you need to have a physician 24 h, 7 days out of seven, in the hospital.*” In phase I trials for rare cancers, the participants are patients, meaning that they are ill and require much more care than a healthy volunteer.

## Methodology

### Number of Patients

The number of participants varies widely for early phase clinical trials in cancer. If the trial only focuses on one specific subtype of rare cancer, then the number of patients included is usually not more than six. If the trial includes different types of rare cancer however, then the number of participants can be as high as 40.

### Study Designs

During the interviews it became clear that one of the most commonly used study designs is still the 3 + 3 design. This is typically the study design that is used to find the right dose. A remark that was made by representative 2 of the academic side was that in the dose escalating part of the study, rare cancer patients are usually not involved. Most of the time, they are enrolled into expansion cohorts, commonly used in phase 1 rare cancer clinical trials. “*The expansion part is very often in specific diseases, so that you have a certain understanding, in which disease you would put a certain drug*.” Representative 6 of the academic side mentioned that these expansion cohorts have been changing over the years: they are becoming larger in size. As a result, the phase II trials are sometimes replaced by expansion cohorts. Corroborating this, representative 2 of the academic side made the following observation: *“We have less and less phase II trials and more and more phase I expansions.”*

Representative 5 of the academic side stated that for immunotherapy in particular, this 3 + 3 design is sometimes replaced by the rolling six design.

Basket trial designs are used in phase I clinical trials for rare cancers, although they can also be implemented into the other phases of clinical development. These trials select patients based on their tumor characteristics and include all types of cancers, both common as well as rare ones. Representative 4 of the academic side claimed that this study design is slowly replacing the “all-comers approach” (i.e., no or very few restrictions on the type of tumors included in the trial). The basket trial was considered a huge improvement over such past trial designs by the interviewees. To illustrate this, representative 4 of the academic side made the next remark: “*Until a couple of years ago we used the ‘all-comers trial’. You just had a trial that does not specify which tumor type of patients could enter. So, we were using the power of serendipity, just coincidence, to find certain correlations*.” This serendipity is now partly excluded because clinicians are gaining much more knowledge about which patients are going to respond by examining their underlying mutations.

The umbrella trials are also becoming more popular. Again, these trial designs are not exclusive to phase I clinical studies and can be used in all other phases of clinical development as well. Umbrella trials are especially relevant for rare cancers because they can tackle the problems inherent to rare diseases since they include all types of mutations within a certain cancer.

However, representative 2 of the academic side noted that these two study designs necessitate a shift in the recruitment strategy of these trials. More specifically, the mutation that causes the cancer needs to be identified before the patient can enter the trial. “*You have the definition of your rare cancer based on the incidence, that is calculated based on the histology. But this study design is based on genomics*.”

Bayesian methods are also used more often. They can considerably speed up the conduct of an early phase rare cancer clinical trial. This was mentioned by several representatives of the academic side. Representative 6 explained this as follows: *“Bayesian designs are accelerated designs, where for example, there is an acceleration within the patient. If there are no signals within the patient, using a Bayesian design, the dose can be escalated more quickly.”*

Representative 4 of the academic side stated that the platform trials are starting to be used more frequently. Their main advantage is the possibility to change the investigational drugs and/or targets. However, the continuous analysis of the data necessary to determine which new drugs can be introduced into or excluded from the trial is accompanied by a large organizational burden.

### International Collaboration

There was some disagreement among the participants regarding the extent to which international collaboration occurs for First-in-Man clinical trials in rare cancers. Some interviewees stated that it happens regularly. For example, representative 2 of the academic side made the next statement: “*I’m not aware of any clinical trials for rare cancers that are specific to only one country. For me it’s not reasonable. So these phase I trials have at least two countries and this can go as high as 10 countries in phase I.*” Other participants claimed the opposite, including representative 3 of the commercial side: *“I don’t think that cooperation happens so much. I think usually for phase I you try to limit it to one or two centers.”* When the answers were examined in more detail, it can be concluded that all the academic representatives mentioned that international collaboration happens, while most of the commercial representatives stated the opposite, i.e., that it was not common practice to collaborate across borders.

The understanding of what exactly constitutes international collaboration also varied among the interviewees. For some of them, collaboration implied that they would refer patients to other centers. Others believed that this meant that multiple centers in different countries are working on the same trial. The latter type of collaboration is mainly organized by pharmaceutical companies. Representative 7 of the academic side stated the following: “*The international cooperation is mainly set up by the pharma, because they organize all the regulatory aspects for the separate countries*.”

One point of agreement among all the interviewees is that the main advantage of research collaborations is that they allow the investigators to tackle the challenges associated with the rarity of the diseases under investigation. Representative 2 of the academic side stated the following: “*The biggest challenge for these clinical trials is to find the patients, because they have rare tumor types. And it is impossible to do without international collaboration.*” International reference centers need to be established to which all patients with a specific rare disease can be sent. It is considered more valuable to have one study with five patients than five studies with one patient.

With respect to the type of international collaboration where multiple centers are working on the same trial, in this situation the sharing of information in order to compare different approaches was also deemed very important by the interviewees. Representative 6 of the academic side stated the following about this subject: “*If a patient gets treated in the Netherlands, and a patient gets treated in France, on paper they may all be the same. But the reality is different*.” It is important to foster an environment in which information and methods can be shared between these settings. However, representative 4 of the academic side stated the following about this type of collaboration: “*I am reluctant to do it, because it is such an intensive approach and a financially intensive way of performing trials*.”

During the interviews, it became apparent that there is also some degree of competition between the centers. Doctors do not want to lose their patients by referring them to another center or country where the clinical trial is being performed. Efforts to harmonize the conduct of phase I trials in rare cancers may also undermine the competitive advantage some countries have as hosts of clinical research activities. For Belgium for example, an interviewee from that country did not want to sacrifice the short timelines discussed above: “*If Belgium is no longer involved in approving the project, because it is in another country, then it can take up to 60 days and this comparing to the 14 days for phase I trials that we now have, is of course troubling*.” It is therefore important to ensure that harmonization efforts do not slow down the conduct of phase I clinical trials in rare cancers at the country level.

### Financial Aspects

Many interviewees stated that a phase I clinical trial, being only a part of the complete clinical research package, is very expensive. The cost of such trials is not only determined by the cost of treating the patient, but also the organizational activities behind the study, the manufacturing of the investigational product etc. As mentioned above, the shift towards new trial designs implies that more trials will have to be initiated to treat the same number of patients. “*Less and less patients are enrolled in one clinical trial and then the total setup cost for clinical trials will become higher, because you have less patients (per trial),*” representative 2 of the academic side argued.

Another financial challenge that was mentioned by the representatives from the academic side is to get pharmaceutical companies interested in sponsoring these clinical trials. Representative 7 of the academic side claimed that “*up till now it was mainly the big tumor groups pharma was interested in. Because those where the groups to which they could sell their products in the end.*” This interviewee also mentioned that clinicians can play an important role in trying to raise the interest of pharmaceutical companies: “*we need to talk to them and express the medical needs. You have examples of successful stories like imatinib in GIST (gastrointestinal stromal tumor), which is a rare cancer. And there was nothing for that, but imatinib is now the first line treatment of choice.*”

### Patients’ Perspectives

#### Accommodation

There was a strong consensus among the interviewees that the accommodations of commercial and academic-sponsored phase I rare cancer trials were very similar. If the subjects have to stay overnight, they will be assigned a hospital room ensuring close proximity of experienced clinicians. Patient organisation representative 2 stated that the trial should be conducted in a single-person room because patients need privacy. Patient organisation representatives 1 and 3 on the other hand believed that the possibility of conducting the trial at home would be more convenient for the patients.

#### Financing

Two of the four interviewed patient organisation representatives were not certain whether or not patients receive compensation for their participation in early phase trials for rare cancers. Regardless, all representatives believed that patients would not take part out of any financial motivations, but because the trial could give them access to a potentially beneficial drug. One representative from the academic setting remarked that they want to avoid situations where cancer patients participate in a phase I trial because of the accompanying monetary compensation. Commercial setting representative 2 mentioned that patients receive monetary compensation to cover the costs they face as a result of participating in the trial.

#### Follow-Up

All patient organisation representatives believed that the follow-up during the trial is very well organised and accurately documented according to the study protocol. According to one representative, patients would prefer to be more involved in the process and submit data and/or comments themselves *via* apps. Some experts representing the commercial setting emphasised that it is crucial to continue providing care to the patient even after the trial has ended, for example, by applying a roll-over protocol which enables patients to continue receiving the treatment within the context of a new research question. Patients can also be referred to different trials, potentially even organised by a different company. The participants can also keep on receiving the investigational drug in case they experience any benefit from it.

#### Administrative Burden

According to two of the patient organisation representatives, patients are confronted with lots of paperwork and many different documents as part of their participation in phase I rare cancer trials, such as summaries of the trial protocol. Two of the patient organisation representatives believed that many patients do not experience this paperwork as burdensome, since they did not think patients read these forms very thoroughly. One patient organisation representative felt that patients do not have enough time to read through the entire information sheet and do not fully understand what is written in this document.

### Recruitment

#### Recruitment Through Hospitals

Two experts, representing the academic setting, mentioned that participants are recruited by clinicians in the participating hospitals directly. Similarly, two representatives of the commercial setting said that the treating physician is mostly responsible for the recruitment of patients for early phase cancer studies. Most patient organisation representatives also confirmed that the recruitment of patients takes place in the hospital by their treating physicians.

#### Recruitment Through Networks

One expert representing the academic setting mentioned that most participants of early phase rare cancer trials are referred to their hospital by colleagues from other hospitals. Another academic setting representative stated that they recruit patients using the networks they established together with smaller hospitals. This expert wanted to emphasize that it is regrettable there are only two European-wide networks for rare cancers (EURACAN and PaedCan) because these are very much needed for ultra-rare tumours. A third expert representing the academic setting remarked that in Netherlands, 14 centres for juvenile melanoma cooperate intensively. Patients can be referred to one of these centres by their oncologist, ophthalmologist and sometimes their general practitioner.

#### Recruitment Through Patient Organisations

All representatives of patient organisations stated that their members share experiences about early phase clinical trials. This can stimulate other patients to participate. Only one patient organisation representative mentioned that patients consult them asking if there are any trials they can participate in. Three of the academic setting representatives said that for the recruitment of participants for First-in-Man rare cancer trials they did not yet work together with patient organisations. They hoped that those patient organisations would inform patients of the existence of such trials to convince them to participate.

#### Motivations for Participating

The patient organisation representatives and the experts from the commercial and academic settings all echoed the same sentiment: “If it is not helpful to the patient, it may well be helpful for other patients with the same disease.” Every interviewee mentioned that a lack of available treatments is one of the main reasons to participate.

### Role of Patient Organizations

One commercial setting representative believed that all large companies are now looking into how they can involve patient organisations to help them set up a protocol addressing patients’ needs. Another expert representing the commercial setting claimed that local patient organisations are not always being involved due to the global nature of their companies’ studies. Commercial setting representative 2 claimed that their company is conducting patient-centric remote trials, whereby patients do not have to visit the hospital at all. According to representatives of both commercial and non-commercial settings, the involvement of patient organisations provides an added value to phase I trials and can improve the design and feasibility of the trial and the legibility of the study documents. Patient organisation representatives additionally mentioned that in recent years, there have been stories about trials going wrong and that the public often thinks that the participating patients were not adequately informed of the risks of the study, but these are only exceptional cases. Usually, the majority of the patients are pleased about the way a trial is executed. In case the trial procedures are too complicated or too burdensome, patients might drop out, severely complicating the conduct and analysis of the study. Medicines that can be applied at home are preferable, but this is not always possible.

### Evolution Over the Next 10 years

#### Rise in Amount of Studies

Due to the increasing development of personalized medicines, there are much more target pathways that can be tested. This testing is usually done in a phase I clinical trial. This is why the amount of early phase studies in rare cancers in all likelihood is going to rise over the next years. Additionally, due to the emergence of new study designs, more studies will have to be initiated to treat the same amount of patients as before.

#### More Collaborations

In the future, there will likely be much more centralization of clinical trials through the setup of international collaborations. Representative two of the academic side stated: *“I think networks will be key, so opening different trials in different centers and just sending the patients to the right trial.”* Within the European Reference Networks EUROCAN for rare adult solid tumor cancers and PAEDCAN for pediatric oncology are installed.

#### Other Study Designs

The used study designs will keep changing more and more to basket and umbrella trials. Platform trials will also become more popular due to the possibility of plugging in different kinds of therapeutic entities. So, the interviewees expect a shift toward more intelligent and adaptive study designs.

#### More Targeted Treatments

Nowadays, whole genome sequencing can be performed. As a result, much more information is available for making predictions on whether patients are going to respond to a treatment or not. In the future, increasingly targeted treatments, more tailored to the patient, will be investigated in phase I trials. The treatments themselves are also going to change. The interviewees predicted that there will be a strong increase in the development of immunotherapies and gene therapies.

## Discussion

### The Organization of Early Phase Clinical Trials for Rare Cancers

First-in-man clinical trials for rare cancers are mostly set up through collaborations between the academic and the commercial side. Clinicians in academic hospitals have much more experience with rare cancer cases but the commercial side was not interested in rare cancers until the introduction of the Orphan Drug Directive (EC 141/2000), since this did not represent a large enough target market for selling their products. However it is not possible to support this conclusion with literature as no specific data can be found about this subject.

### Financing of Early Phase Clinical Trials for Rare Cancers

Compared with the available literature concerning early phase clinical trials for common cancers (Chakiba et al., 2018), a notable difference is observed in the amount of industry sponsored trials. In common cancers, 53% of the phase I clinical trials are sponsored by industry, while this paper concludes that the vast majority of rare cancer trials are industry-sponsored. An explanation for this difference can be found in some of the statements that the representatives of the academic side made. They stated that they were not able to properly initiate international collaborations themselves because they could not organize the regulatory aspects in the different countries, and that these were always set up by the commercial sector. As described in the section above, first in man clinical trials for rare cancers require international collaborations to tackle the problem of rarity. Therefore, it makes sense that academic centers cannot set up these trials by themselves and that this mainly has to be done by pharma companies. However, there is no literature available to corroborate this potential explanation.

Though, the article by Kummar Kakkar et al. (Kumar Kakkar and Dahiya, 2014) presents another explanation. It is seen that more and more pharmaceutical companies are becoming interested in developing drugs for rare diseases because of the benefits associated with the Orphan Drug Regulation (e.g., short clinical development timelines, market exclusivity for 10 years, etc.). On top of these benefits, the article of Attwood et al. (Attwood et al., 2018) made the remark that 29% of these orphan drugs now have large patient populations and thus have high profit margins and several authorized orphan drugs for rare cancers got multiple rare cancer indications (Dooms, 2017). The combination of these benefits and high profits can be an explanation for the higher amount of pharma sponsored trials in rare cancers than in common ones. The average cost to execute these trials roughly lies between the 10.000 and 50.000 euros per patient. It is however difficult to generalize this estimation because it depends on what specifically is requested within the procedure. The patients are compensated for the costs associated with their participation (lunch—travel) but never rewarded, as this is considered not ethical.

## Methodology of Phase I Clinical Trials

When the available literature on the methodology of phase I trials in general is consulted (Wong et al., 2016), a strong convergence with the conclusions concerning the designs of early phase rare cancer trials can be observed. While new trial designs have been developed over time, the classical 3 + 3 design remains the most used design in phase I rare cancer trials. According to Wong et al. (Wong et al., 2016), this is mainly because clinicians have not yet fully mastered the use of such new study designs, and because their novelty complicates the approval of the trial protocol by ethics committees. Additionally, they also necessitate better communication between the sites. This undermines the many benefits (Manji et al., 2013; Wong et al., 2016) of these new designs. Although the article by Wong and colleagues (Wong et al., 2016) focuses on common cancers, its conclusions likely apply to first in man rare cancer trials as well.

The use of expansion cohorts was also mentioned. During the interviews, it was observed that this is still controversial: some participants fully support these, while other participants think their use should be limited. In the available literature (Manji et al., 2013), it is mentioned that the use of expansion cohorts is rising but that there is still no consensus about when they should be introduced into a trial. Their major advantage is that they could be used to determine the efficacy of the drug early on and to therefore minimize the need for a phase II study to be performed. These benefits were also brought up by representative 6 of the academic side, who highlighted the negative aspects of these cohorts as well. For example, expansion cohorts often lack statistical power due to the limited number of patients included. This is also mentioned in the literature (Manji et al., 2013).

### International Collaboration

The need for international collaboration in early phase clinical trials for rare cancers is clear, both from the interviews and from the available literature (Gatta et al., 2011), as they remain the only feasible solution to tackle the issue of rarity. However, this international collaboration is also accompanied by some challenges mainly by different national regulations.

The challenges mentioned by the interviewees are corroborated by the available literature. For example, it is difficult to come to a consensus when multiple different countries are involved (Komatsubara and Carvajal, 2016). Furthermore, it is also important that incentivizing policies instituted by individual countries with respect to the conduct of trials remain in place.

### The Evolution of Early Phase Clinical Trials for Rare Cancers

It was mentioned that there will likely be a rise in the number of phase I rare cancer trials in the coming years due to the evolving science behind cancer (i.e., more tailored medicines, more rare cancers due to more detailed genome sequencing). When there is already treatment available, the pharmaceutical industry will not be eager to invest. This conclusion is certainly substantiated in the literature (Blay et al., 2016; Boyd et al., 2016; Komatsubara and Carvajal, 2016).

### Patient Organization Representatives

Sufficient understanding about phase I clinical trials was hard to find in this group of participants and they were not aware about the academic and/or commercial setup of the study. Ambiguity about financial costs/compensations can be explained by the fact that these patients are terminally ill and regularly hospitalized. A financial compensation (travel and food) was generally considered inferior to a possible positive effect in treatment. Insurances for clinical trials never compensate the patients. Concerning the follow-up, Hutchison (Hutchison, 1998) could demonstrate that nursing and clinical care/attention in phase I cancer studies was mostly experienced by subjects as “very good” or at least “just right”.

According to two of the patient representatives, the participants have to deal with lots of paperwork and the informed consent does not seem to be read thoroughly. This was also reported by Hutchison (Hutchison, 1998) who confirmed that patients were not always interested in all the details of the study.

### Recruitment of Subjects

One center will never have sufficient patients for this type of study and needs to collaborate, sometimes internationally, as mentioned by one academic and Fox et al. (Fox et al., 2017).

Financing seems to be the main hurdle but pharmaceutical companies can bring in some funding besides their international network. Also governmental arrangements and a strong collaboration with international trial groups needs to be achieved (Fox et al., 2017). Mandrekar et al. (Mandrekar et al., 2015) and all our patient representatives confirmed that participation in early clinical trials for rare cancer was experienced as positive and stimulating to convince other participants as the ineffectiveness of other treatments was the main stimulus (Dolly et al., 2016; Catt et al., 2011).

### Role of Patient Organizations

All patient organization representatives indicated that they would like to be involved more but that they were not aware of all the different first in human studies. Organizations like the Patient Focused Medicines Development initiative (https://patientfocusedmedicine.org/about-pfmd/) can assist in the design and the development of research and medicines by focusing on unmet patient needs. Moreover, patients would like to help in increasing the legibility of the informed consent form (34). No data could be found and no participants expresses any preference for academic nor commercial studies.

## Limitations

A major limitation of this study is the small number of participants. Only 15 participants were interviewed, despite 87 experts contacted. This is a very low response rate (17.2%), and data saturation was therefore not reached.

Another limitation is that experts from only five different European countries were included. As a result, the participants are not a good representation of the target population and caution should be taken when these results are generalized to the broader European setting. Further research is necessary to fully support this generalization.

## Conclusion

Representatives of the academic and the commercial sites collaborate in the majority of early phase rare cancer clinical trials: the commercial partner finances the trial, whereas academia is responsible for the execution of the procedure of the trial. A very limited budget is available to execute pure academic studies for rare cancers.

Audits and inspections are conducted in the premises executing these trials, but the frequency and setup varies widely. The inspections are mainly organized by the national health authorities and they take place between once every year and every 3 years. The internal audits are organized by the company/hospital itself and happen very frequently. The external audits are organized by the sponsor, and their intensity is directly proportional to the recruitment of the trial. Belgium has short timelines, the possibility to ask for advice and strict but correct regulations. However, also some negative aspects were mentioned, like the difference between different ethics committees, the over-interpretation of the rules, the insufficiently available qualified personnel and the high burden for them.

Research into the methodology of phase I clinical trials for rare cancers revealed that the 3 + 3 design remains the most widely used design and that the use of expansion cohorts remains controversial.

During the interviews, the importance of international collaboration was emphasized, as this is the best approach to tackle the issue of rarity. However, a more centralized approach needs to be balanced with efforts to incentivize clinical research on the national level.

Patients experience no differences between academic and commercial early phase clinical trials nor in participation nor in transport to the setting nor in follow-up. Patient organizations may contribute in recruitment, feasibility and legibility of the informed consent forms.

Finally, the growing need for first in man rare cancer trials is high, not only because rare cancer patients deserve the best treatment, but also because medicines developed for the treatment of rare cancers represent the future for cancer therapy in general.

## Data Availability

The raw data supporting the conclusion of this article will be made available by the authors, without undue reservation.

## Abbreviations

CAR-T cell, Chimeric antigen receptor T-cell; CRM, Continual Reassessment Method; CT, Computerized tomography; DLT, Dose Limiting Toxicity; EMA, European Medicines Agency; EU, European Union; IBRD, International Bootcamp Rare Diseases; ICF, Informed Consent Form; IP, Investigational Product; GIST, Gastro Intestinal Stromal Tumor; KCE, Federaal Kenniscentrum Gezondheidszorg; MRI, Magnetic resonance imaging; MTD, Maximum Tolerated Dose; RARECARE, The project Surveillance of Rare Cancers in Europe; RCE, Rare Cancers Europe.
