# Supplementary material for: Characteristics of Early Phase Clinical Trials for Rare Cancers: Insights From Interviews With Stakeholders
Source: Front Pharmacol. 2022 May 2;13:775217. doi: 10.3389/fphar.2022.775217 (PMC9108391; doi:10.3389/fphar.2022.775217)
Supplement: Supplementary file 1 [file DataSheet1.PDF]

Phase I clinical trials for rare cancers

Nodes

Name	Description	Files	References
Challenges		0	0
Amount of patients		7	9
Solution		4	4
Challenge of study designs		4	7
Challenge of expansion cohorts		2	2
Challenges of basket trials		1	2
Change of definition		1	1
Rarity		1	2
Competition		2	2
Cooperation challenges		8	16
Different approaches		3	3
Different regulatory environments		2	2
Financial side of cooperations		4	6
Lack of information sharing		1	1
Lack of networks		1	1
Loss of benefits		2	3
Drug approval		2	2
Expectations		2	3

Name	Description	Files	References
Financial challenges		5	7
High costs of trials		2	3
Interest of pharma		3	3
Solutions		1	1
Harmonization		2	3
Never ending story		1	1
Rare becomes more rare		4	7
Evolution over 10 year		0	0
Amount		2	3
Communication		1	1
International		2	2
More targeted research		5	9
Other endpoints		4	4
Study designs		5	5
Financing		0	0
Mechanisms		10	15
Financed by Academia		2	2
Financed by other organisations		2	2
Financed by Pharma		10	12
Patient compensations		0	0
Healthy volunteers		2	2
Particapation compensation		10	10
Reason behind		5	5
Travel compensation		9	12

Name	Description	Files	References
The needed budget		10	10
Methodology		0	0
Cooperations		0	0
Oppurtunities		6	10
Experience		1	2
Recruitment		5	7
Organisation		8	11
Organised by networks		2	3
Organised by Pharma		4	4
Way of execution		4	4
The extent		8	12
Number of participants		10	11
Organisation of trial		5	5
Used study designs		0	0
3 + 3 cohorts		5	6
Basket		4	4
Bayashian		2	2
Now		10	21
Other study designs		7	9
Past		2	2
Umbrella		5	6
Patients		0	0
Accomodation		15	16
Awareness of setting		0	0

Name	Description	Files	References
Patient organization		3	5
Patient themselve		2	2
Financing		0	0
Compensation for the trial		3	3
Sufficient		4	4
Compensation for transport		1	2
Compensation from insurance		1	1
Particapation compensation		10	10
Follow-up		0	0
After the trial		8	8
During the trial		13	16
Involvement		8	9
Added value		0	0
Feasibility		7	16
ICF		7	13
Information		5	7
Support participation		4	4
What is important for the patient		5	6
Illusion		4	7
Motivation		0	0
Altruism		4	4
Lack of treatment		6	7
Last hope		9	13

Name	Description	Files	References
New treatment		3	3
Setting		15	19
Recommendation of patient organization		4	4
Recruitment		0	0
Hospital		7	8
Networking		8	10
Own initiative		1	1
Patient organizations		7	9
Regulation		0	0
A lot of paperwork		2	3
Burden		2	3
Not enough time		1	1
Remarks on the setting		0	0
Academic		0	0
Commercial		1	1
General		3	3
Regulation		1	1
Audits and inspections		0	0
External		7	9
Internal		6	7
Negative aspects		0	0
Ethical commitees		2	3
Informed consent form		2	3

Name	Description	Files	References
Interpretation		1	1
Lack of advice		2	2
Resources		4	7
Positive aspects		8	11
Advice		2	2
Experienced persons		2	2
Rules		2	2
Short timelines		4	4
